# Correction to: General conditions for Turing and wave instabilities in reaction–diffusion systems

**DOI:** 10.1007/s00285-023-01884-x

**Published:** 2023-03-21

**Authors:** Edgardo Villar-Sepúlveda, Alan R. Champneys

**Affiliations:** Engineering Mathematics, University of Bristol, Ada Lovelace Building, Tankard’s Cl, University Walk, Bristol, Somerset BS8 1TW UK

**Correction to**: **Journal of Mathematical Biology (2023) 86:39** https://doi.org/10.1007/s00285-023-01870-3

In the original publication of the article, the article title was published incorrectly. The correct title is provided in this Correction.

The original article has been corrected.

